# A Case of Fluorosis: Fluoride-Induced Osteopetrosis

**DOI:** 10.7759/cureus.16479

**Published:** 2021-07-19

**Authors:** Abdul Hakim Almakadma, Sami Almustanyir, Hosam Aldalbahi, Suphia M Sherbeeni, Omar AlHuzaim

**Affiliations:** 1 College of Medicine, Alfaisal University, Riyadh, SAU; 2 Department of Medicine, Ministry of Health, Riyadh, SAU; 3 Medical Specialty Department, King Fahad Medical City, Riyadh, SAU; 4 Internal Medicine, Tadaw Medical Complex and Day Surgery Center, Riyadh, SAU; 5 Obesity, Endocrine and Metabolism Center, King Fahad Medical City, Riyadh, SAU

**Keywords:** fluorosis, osteopetrosis, fluoride, bone mineral density, musculocutaneous

## Abstract

There are multiple etiologies of increased bone density, including osteopetrosis and fluorosis. Osteopetrosis can either be a malignant autosomal recessive condition found in children or a benign autosomal dominant adult variant; both of which are characterized by decreased bone resorption. In contrast, fluorosis is characterized by increased bone formation secondary to chronic fluoride intoxication, but with a similar clinical manifestations to osteopetrosis.

A 70-year-old lady with generalized joint aches, stiffness as well as fatigue, was found to have high bone mineral density and alarmingly high fluoride levels. The patient was found to be drinking fluoride containing water from an untreated local well for many years.

Fluorosis results in increased bone mineral density and disease progression correlates with length of exposure. Fluorosis can result in reversible musculocutaneous symptoms and radiological findings. However, severe chronic cases may develop irreversible neurologic manifestations. Urinary fluoride testing is the screening modality of choice, and the key component of management is avoidance of the source of fluoride intoxication as well as monitoring of urinary fluoride levels.

## Introduction

An important acquired osteopetrosis like disease is fluorosis. Osteopetrosis is a rare sclerosing inherited dysplasia of bone [1], which results in an increased tendency towards fractures with minimal effect on bone healing. In all forms of osteopetrosis, the underlying pathophysiology is impaired osteoclastic function resulting in dense, deformed sclerotic bones [2]. The more severe forms tend to occur in infancy and have an autosomal recessive inheritance with an incidence of 1 in 250,000 births. Mild forms are observed in adults and are inherited in an autosomal dominant manner with an incidence of 5 in 100,000 births [3,4]. Diagnosis is largely based on clinical and radiographic evaluation and can be confirmed with genetic testing where applicable. Unlike osteopetrosis, fluorosis is characterized by increased bone formation secondary to chronic fluoride intoxication. Fluorosis presents with a similar clinical and radiological manifestation to osteopetrosis, as discussed in our case. Therefore, distinction between osteopetrosis and fluorosis is imperative.

## Case presentation

A 70-year-old female from Ad Dawadmi, a town in the Riyadh province, presented with past medical history of hypertension, dyslipidemia, poorly controlled insulin dependent diabetes mellitus type II for nine years, complicated with non-proliferative diabetic retinopathy as well as diabetic nephropathy. Furthermore, our patient is known to have history of fragility fractures in the right femur 20 years ago followed by right tibia and fibular fractures post open reduction and internal fixation (ORIF) seven years ago after taking alendronate for one year, which was then discontinued and is currently on vitamin D 1000 IU daily.

Our patient first presented to our clinic in 2010 in a wheelchair with complaints of generalized bone pain, more prominent in the back as well as generalized fatigue. Vitals were stable. A thorough history revealed that our patient has been drinking water from an untreated local well for years. Physical examination did not yield any significant findings. Blood fluoride levels were sent immediately and revealed a value of 7.9 mcmol/L (normal value: 0-4). Laboratory results including bone profile and renal workup are provided in Table 1.

**Table 1:** Laboratory Results PTH: Parathyroid Hormone; ACR: Albumin-to-Creatinine Ratio; eGFR: Estimated Glomerular Filtration Rate; LDL: Low-Density Lipoprotein; HDL: High-Density Lipoprotein.

Laboratory Parameters	Result	Renal Workup	Result	Other Labs	Result
Calcium	2.02 mmol/L	Creatinine	101 umo/L	HBA1c	10
Phosphate	Normal	ACR	51 mcg/L	LDL	1.7
Vitamin D	Normal	eGFR	44	HDL	0.9
PTH	Normal	Renal U/S	9.3 cm bilateral simple cyst		

**Figure 1:**
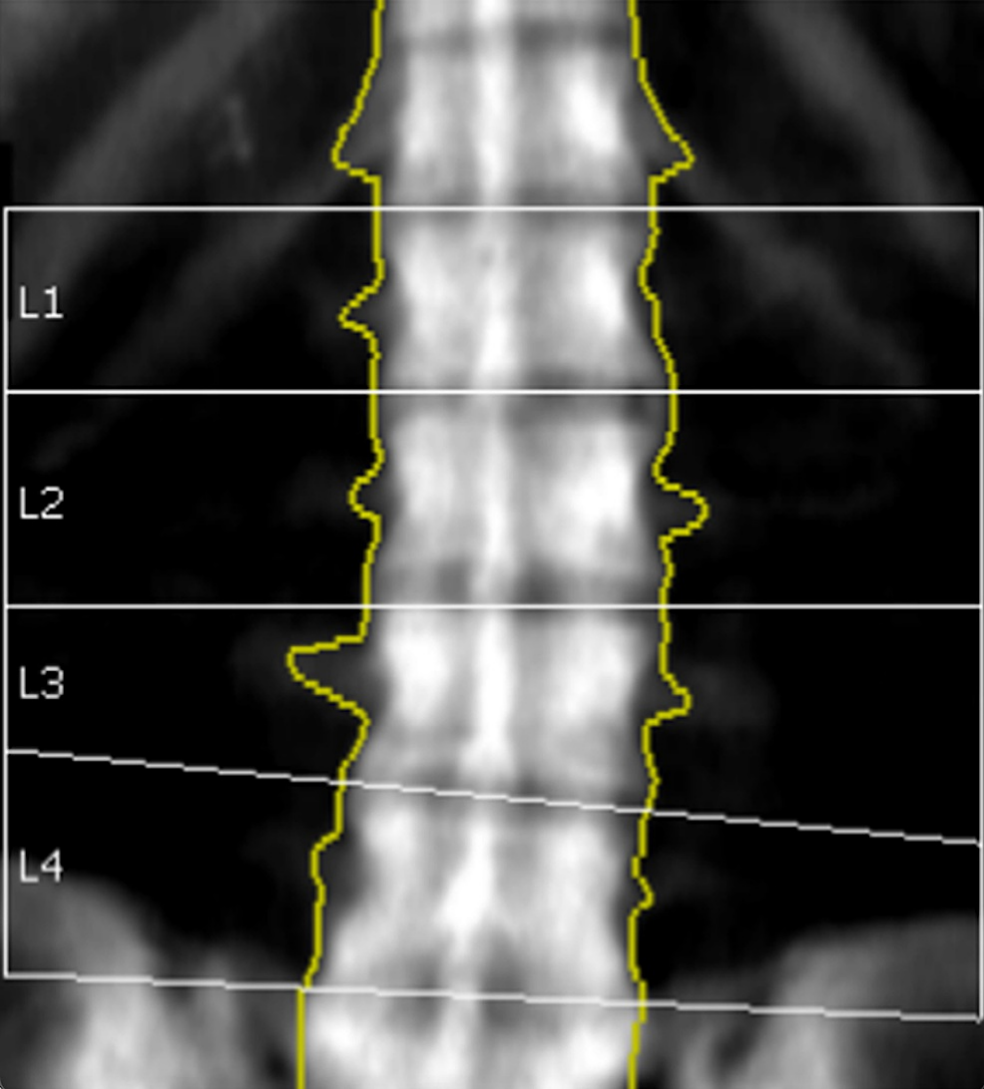
Initial BMD of Lumbar Spine (2010). BMD: Bone Mineral Density

**Figure 2:**
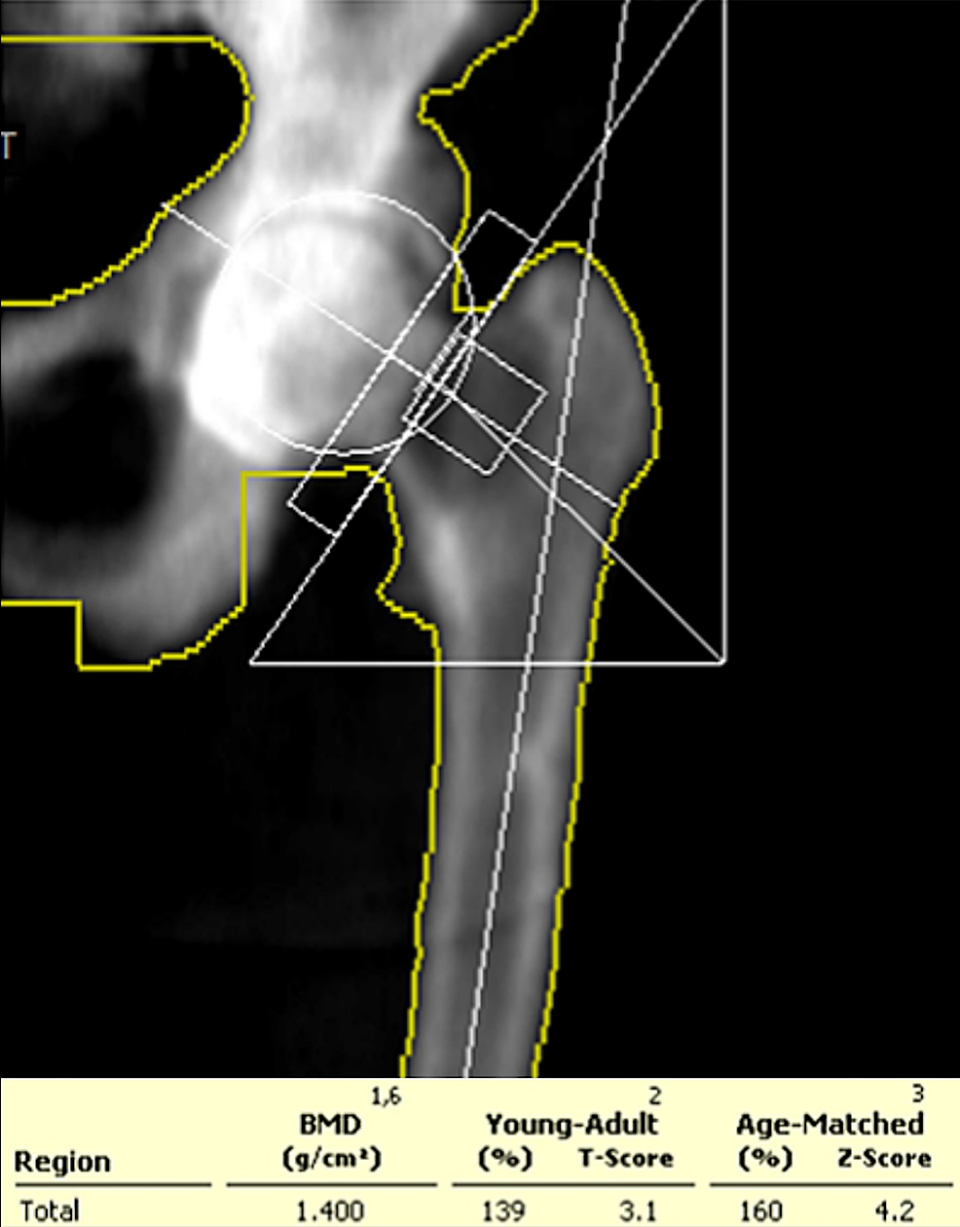
Initial BMD of Left Femur (2010). BMD: Bone Mineral Density

**Figure 3:**
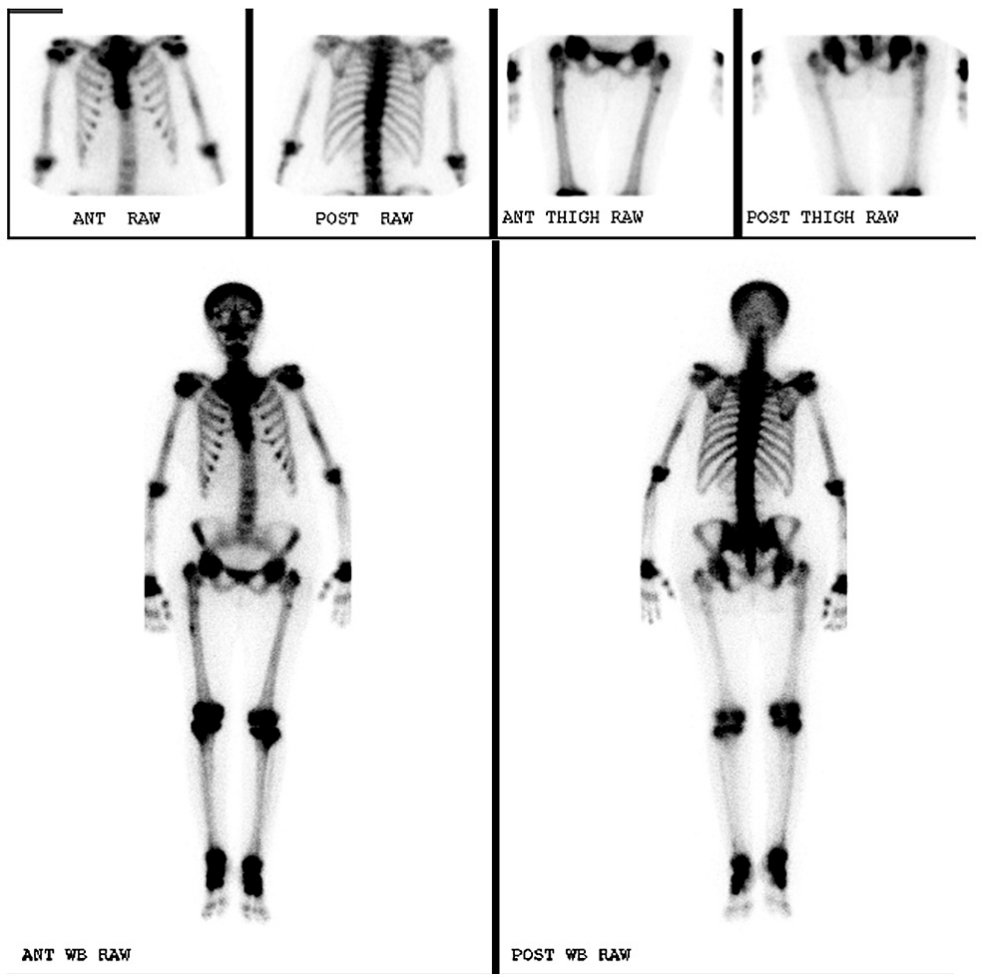
Whole Body Bone Scintigraphy.

An initial dual energy X-ray absorptiometry scan was performed for both lumbar spine and LEFT femur neck using a lunar prodigy advanced machine in 2010 and bone mineral density (BMD) results were obtained according to NHANES reference populations. Initial findings showed a BMD of the lumbar spine (L2-L4) was 2.609 g/cm², which is 13.1 standard deviations above the mean for age matched persons and equivalent to T-score of +11.7, as illustrated in Figure 1 and Table 2. Furthermore, a BMD of the total hip of the LEFT femur was 1.400 g/cm², which is 4.2 standard deviations above expected for her age and equivalent to T-score of +3.1, as illustrated in Figure 2 and Table 3. Furthermore, a TC-99m methylene diphosphonate (MDP) bone scintigraphy was also performed which revealed diffusely increased tracer uptake in the axial and appendicular skeleton with more pronounced periarticular uptake. It also showed an increased bone to soft tissue uptake ratio, as well as small foci of periosteal uptake in the upper femurs, as shown in Figure 3.

**Table 2:** Initial BMD and T-Score of Lumbar Spine (2010). BMD: Bone Mineral Density

Region	BMD (g/cm^2^)	Young Adult T-Score	Age Matched Z-Score	Area (cm^2^)	Width (cm)	Height (cm)
L1	2.579	12.1	13.4	13.16	4.5	2.94
L2	2.494	10.8	12.2	16.4	4.8	3.44
L3	2.645	12	13.4	15.3	5	3.05
L4	2.692	12.4	13.8	16.15	5	3.23
L1-L2	2.532	11.4	12.8	29.56	4.6	6.38
L1-L3	2.57	11.7	13	44.86	4.8	9.43
L1-L4	2.602	11.9	13.2	61.01	4.8	12.66
L2-L3	2.567	11.4	12.8	31.7	4.9	6.49
L2-L4	2.609	11.7	13.1	47.85	4.9	9.72
L3-L4	2.669	12.2	13.6	31.45	5	6.27
T-Score for Vertebral Height (L2-L4):			Compared to Young Adult (T-Score):	-1.36	Adjusted for Stature (T-Score):	-0.27

**Table 3:** Initial BMD and T-Score of Left Femur (2010). BMD: Bone Mineral Density

Region	BMD (g/cm^2^)	Young Adult T-Score	Age Matched Z-Score	Area (cm^2^)
Neck	1.503	3.3	4.7	3.74
Upper Neck	1.417	5	6.2	1.59
Lower Neck	1.568	-	-	2.14
Wards	0.94	0.2	2	3.3
Troch	1.317	4.1	5.1	11.88
Shaft	1.444	-	-	13.45
Total	1.4	3.1	4.2	29.07

**Figure 4:**
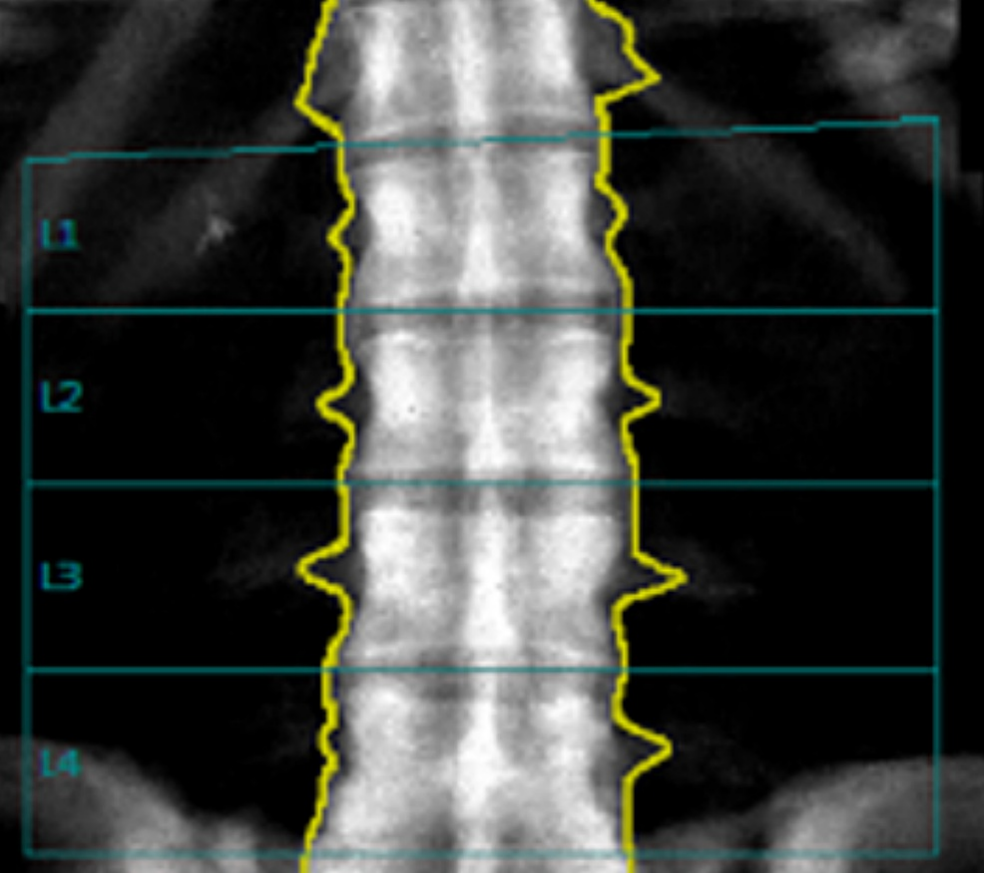
Follow-up Lumbar Spine BMD in 2019. BMD: Bone Mineral Density

**Figure 5:**
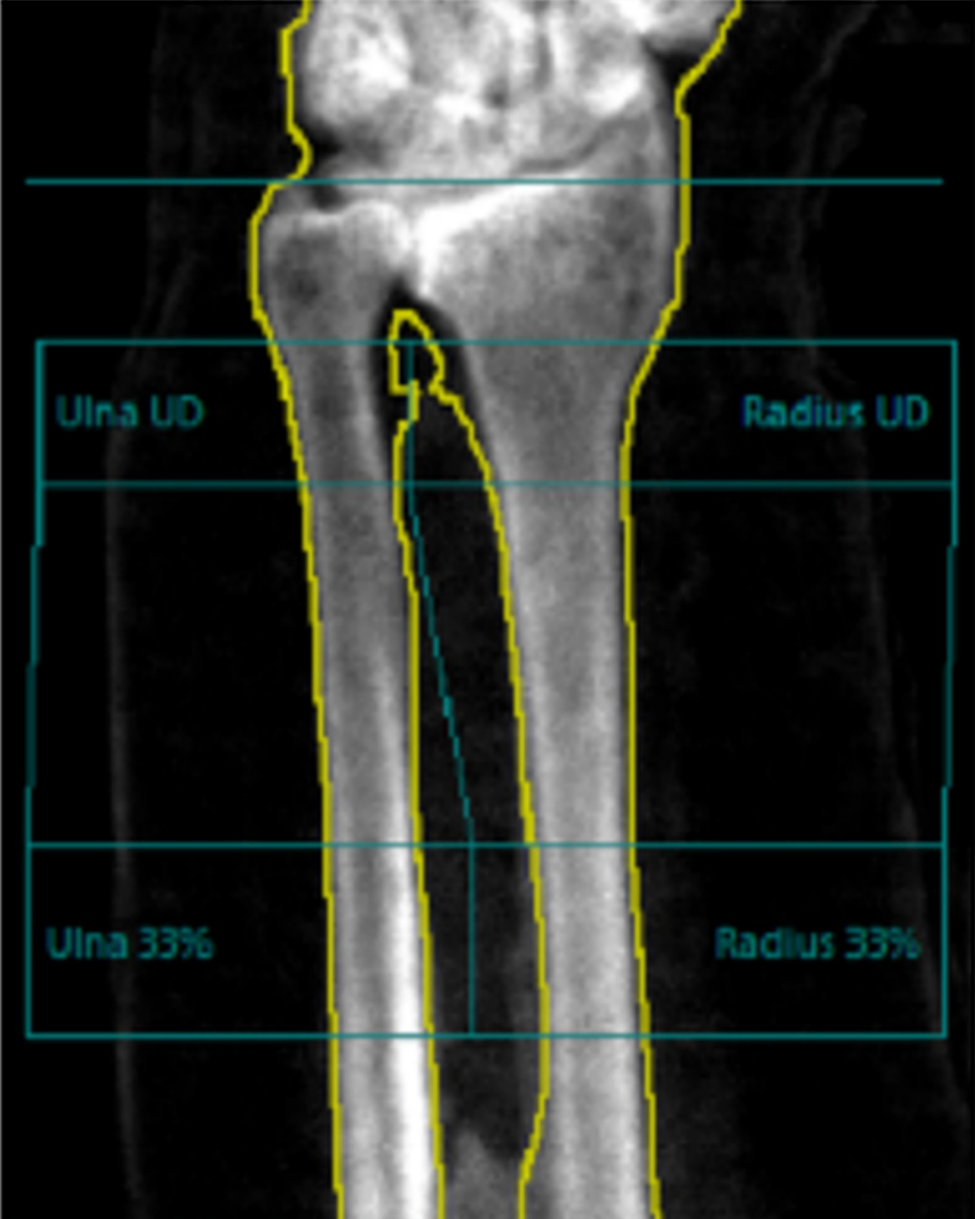
BMD of the Distal Third of the Left Radius. BMD: Bone Mineral Density

Finally, a follow-up bone mineral density in 2019 was performed. The BMD of the lumbar spine (L1-L4) equalled to 2.720 g/cm^2^ which corresponds to a T-score of 12.8 (previously 2.673 g/cm^2^), as illustrated in Figure 4 and Table 4. Furthermore, a comparison between the different BMD of the lumbar vertebrae results is demonstrated in Table 5 showing a continued increase in BMD. The BMD of distal 3rd of left radius equalled to 0.982 g/cm^2^ which corresponds to a T-score of 0.6 (previously g/cm^2^ of right femur), as demonstrated in Figure 5 and Table 6. The right femoral head/neck cannot be used for BMD estimation due to previous history of fracture and fixation.

**Table 4:** Follow-up BMD and T-Score of Lumbar Spine (2019). BMD: Bone Mineral Density

Region	BMD (g/cm^2^)	Young Adult T-Score	Age Matched Z-Score	Area (cm^2^)	Width (cm)
L1	2.639	12.6	14.2	13.66	4.5
L2	2.686	12.4	14	14.96	4.8
L3	2.748	12.9	14.6	16.73	5
L4	2.788	13.2	14.9	16.87	5.1
L1-L2	2.663	12.5	14.2	28.62	4.7
L1-L3	2.695	12.7	14.4	45.35	4.8
L1-L4	2.72	12.8	14.5	62.22	4.9
L2-L3	2.719	12.7	14.3	31.69	4.9
L2-L4	2.743	12.9	14.5	48.56	5
L3-L4	2.768	13.1	14.7	33.6	5.1
T-Score for Vertebral Height (L2-L4):		Compared to Young Adult (T-Score):	-1.4	Adjusted for Stature (T-Score):	-0.31

**Table 5:** BMD Trend Over Time. BMD: Bone Mineral Density

Measurable Date (mm/dd/yyyy)	Age (years)	BMD (g/cm^2^)	Change vs. previous (g/cm^2^)	Change vs. previous (%)
1/20/2010	60.5	2.602	-	-
12/31/2012	63.6	2.673	0.071	2.7
4/14/2019	69.8	2.72	0.047	1.8

**Table 6:** BMD and T-score of the Distal Third of the Left Radius. UD: Ultra Distal; BMD: Bone Mineral Density.

Region	BMD (g/cm^2^)	Young Adult T-Score	Age Matched Z-Score	Area (cm^2^)
Radius UD	0.574	2.5	4.3	2.64
Ulna UD	0.475	-	-	1.65
Radius 33%	0.928	0.6	2.4	2.01
Ulna 33%	0.947	-	-	1.88
Both UD	0.536	-	-	4.28
Both 33%	0.937	-	-	3.89
Radius Total	0.771	1.6	3.4	8.91
Ulna Total	0.705	-	-	7.02
Both Total	0.742	-	-	15.93

The follow-up bone density and T-score results showed a drastic increase in bone density from the initial 2010 bone density scan. Upon re-questioning it was found that our patient was non-compliant with refraining from the consumption of the fluoride containing untreated well water, despite initial management plan and emphasis on its importance. Extensive patient counseling and re-education was put in place to ensure the patient understood that the fluoride contained within the well water is the causative agent to her persistent body aches. It was further explained that the hallmark of treatment revolved around the avoidance of the fluoride containing well water. Furthermore, multiple physicians and family members were involved to ensure appropriate compliance with the management plan as well as compliance with regular outpatient follow-ups for re-assessments and monitoring of disease via fluoride urine measurements.

## Discussion

Osteopetrosis has been known to be a bone disorder associated with an increase in bone mass, similar to fluorosis, a chronic fluoride intoxication, first described in 1932 [5]. Even though both diseases result in increased bone density and less commonly bone fractures, the mechanism by which this occurs helps differentiate the two diseases. Osteopetrosis is due to decreased bone resorption, while fluorosis occurs as a result of increased bone formation. Fluoride acts as a stimulator of osteoblasts, it has been suggested that fluoride can play a therapeutic role in the treatment of low bone density diseases such as osteoporosis for its osteoblastic activity. However, high amounts of fluoride supplementations increase the fracture tendency of bones rather than help maintain its integrity [6, 7]. Fluorosis can be due to fluoride inhalation in occupational exposures such as manufacturing of aluminum, steel, glass and iron [8]; however, it is usually found as a result of increased oral fluoride intake. Levels of >1.4 mg/L have been found to result in classic radiological features of diffuse osteosclerosis with calcification of ligamentous insertion, chalky bone appearance and osteophyte formation. Density of bone correlates with time of exposure to fluoride. Bone changes are most commonly found in the axial skeleton, furthermore, the earliest and most severe radiological changes are seen in the cervical vertebrae as well as the pelvis. Typically, in order for clinical symptoms of fluorosis to manifest, sustained ingestion of fluoride for 10-20 years is required [9]. Symptoms of fluorosis include mainly musculocutaneous involvement, especially in the early course of presentation [10, 11], vague joint aches, stiffness and limited range of motion occurs, followed by kyphosis, flexion contractures of the lower extremities and restricted chest wall expansion in some cases. Lastly, neurologic symptoms may occur, however they are less common and are indicative of advanced disease [12]. The most accurate screening modality for the diagnosis of fluorosis includes a urinary fluoride measurement [13]. Other associated laboratory tests include an increase in alkaline phosphatase. Medical management of fluorosis is centered on the removal of the source of fluoride [8], which has shown reversible outcomes in both skeletal radiological finding as well as symptomatic features despite chronic exposure. However, neurological consequences, if occur, are irreversible. Once the fluoride source is removed, regular monitoring of urine fluoride levels is initiated. It is important to note that fluoride has a half-life of eight years and therefore urinary fluoride levels may remain elevated for years [9].

## Conclusions

Adult variants of osteopetrosis and fluorosis have similar symptoms and radiological findings including an increased bone mineral density. However, the underlying pathophysiology of increased bone density distinguishes osteopetrosis from fluorosis. Osteopetrosis is characterized by decreased bone resorption in contrast to fluorosis which is characterized by increased bone formation. Fluorosis can result in reversible musculocutaneous symptoms and radiological findings and disease progression correlates with the length of exposure to fluoride. However, severe chronic cases may develop irreversible neurologic involvement. Urinary fluoride testing is the most accurate screening modality, and the key component of management is the removal of the source of fluoride intoxication as well as monitoring of urinary fluoride levels.

## References

[1] Stoker DJ (2002). Osteopetrosis. Semin Musculoskelet Radiol.

[2] Carolino J, Perez JA, Popa A (1998). Osteopetrosis. Am Fam Physician.

[3] Stark Z, Savarirayan R (2009). Osteopetrosis. Orphanet J Rare Dis.

[4] Bollerslev J, Andersen PE Jr (1988). Radiological, biochemical and hereditary evidence of two types of autosomal dominant osteopetrosis. Bone.

[5] de Vernejoul MC (1990). Fluorosis, osteopetrosis, and ectopic calcification. Curr Opin Rheumatol.

[6] Meunier PJ, Sebert JL, Reginster JY (1998). Fluoride salts are no better at preventing new vertebral fractures than calcium-vitamin D in postmenopausal osteoporosis: the FAVOStudy. Osteoporos Int.

[7] Rosen CJ (2000). Fluoride and fractures: an ecological fallacy. Lancet.

[8] Grandjean P, Thomsen G (1983). Reversibility of skeletal fluorosis. Br J Ind Med.

[9] Fisher RL, Medcalf TW, Henderson MC (1989). Endemic fluorosis with spinal cord compression: a case report and review. Arch Intern Med.

[10] Singh A, Jolly SS, Bansal BC, Mathur CC (1963). Endemic fluorosis: epidemiological, clinical and biochemical study of chronic fluorine intoxication in Panjab (India). Medicine.

[11] Hodge HC, Smith FA (1977). Occupational fluoride exposure. J Occup Med.

[12] Sauerbrunn BJ, Ryan CM, Shaw JF (1965). Chronic fluoride intoxication with fluorotic radiculomyelopathy. Ann Intern Med.

[13] Zenz C (1988). Occupational Medicine. Principles and Practical Applications. https://onlinelibrary.wiley.com/doi/abs/10.1002/smi.2460050317.

